# Senescence Inducer Shikonin ROS-Dependently Suppressed Lung Cancer Progression

**DOI:** 10.3389/fphar.2018.00519

**Published:** 2018-05-23

**Authors:** Hongming Zheng, Qiuju Huang, Suchao Huang, Xia Yang, Ting Zhu, Wensheng Wang, Haojia Wang, Shugui He, Liyan Ji, Ying Wang, Xiaoxiao Qi, Zhongqiu Liu, Linlin Lu

**Affiliations:** ^1^International Institute for Translational Chinese Medicine, Guangzhou University of Chinese Medicine, Guangzhou, China; ^2^State Key Laboratory of Quality Research in Chinese Medicine, Macau University of Science and Technology, Taipa, Macau

**Keywords:** shikonin, lung cancer, senescence, ROS, DNA damage

## Abstract

Lung adenocarcinoma (LAC), predominant subclassfication of lung cancer, leads high incidence and mortality annually worldwide. During the premalignant transition from lung adenomas to LAC, cellular senescence is regard as a critical physiological barrier against tumor progression. Nevertheless, the role of senescence in tumorigenesis is controversial and few senescence inducers are extensively determined. In this study, we used two classical cell lines A549 and H1299 and two NSCLC xenograft models on Balb/c-nude mice to reveal the pro-senescence effects of shikonin and the corresponding underlying mechanism in LAC. Shikonin, a pure compound isolated from the herbal medicine *Lithospermum erythrorhizon*, remarkably stimulated cellular senescence including increased SAHF formation, enlarged cellular morphology, and induced SA-β-Gal positive staining. Further mechanism study revealed that the pro-senescence effect of shikonin was dependent on the increased intercellular ROS generation, which subsequently triggered DNA damage-p53/p21^waf^ axis without activating oncogenes such as Ras and MEK-1. Meanwhile, Kdm2b, an H3K36me2-specific demethylase effectively suppressed ROS generation, was also notably suppressed by shikonin treatment. Moreover, shikonin at 10 mg/kg significantly inhibited tumor weights by 55.84% and 50.98% in A549 and H1299 xenograft model, respectively (*P* < 0.05) through activating cellular senescence. Our study suggested that shikonin, a ROS-dependent senescence inducer, could serve as a promising agent for further lung cancer treatment.

## Introduction

Lung adenocarcinoma, predominantly developed from pre-malignancy lung adenomas, leads estimate 910,000 incidence and 800,000 mortality annually worldwide (Curado et al., 2008; Ferlay et al., 2015). Pathological study revealed that cellular senescence, an intrinsic and irreversible suicide program, is accelerated in lung adenomas while strikingly diminished in LAC, suggesting senescence is a critical physiological barrier against tumor progression (Collado et al., 2005). Furthermore, during chemotherapy and radiation, a robust of senescence stimulation is also found to synergistically determine cancer prognosis and systemic toxicity. For instance, loss of p14^ARF^-dependent senescence correlated with poor outcome of non-small cell lung cancer (Han et al., 2017). Meanwhile, parallel to apoptosis activation, chemotherapy-induced senescence predominately triggered regenerative response against systemic toxicities including bone marrow suppression and gastrointestinal toxicity (Shao et al., 2013). Due to intrinsic and primary physiological response against tumorigenesis, senescence-targeting approach were estimated to cause fewer feedback signaling alterations (Dörr et al., 2013) compared to other targeted chemotherapeutic agents such as gefitinib, afatinib, and cetuximab (Fertig et al., 2016). Moreover, “synthetic senescent interaction” could be also achieved by combining *Kras* activation with *Cdk4* loss, *Myc* stimulation with *Cdk2* depletion (Campaner et al., 2010; Puyol et al., 2010). Therefore, pro-senescence therapy is regarded as a novel and promising strategy for cancer eradication.

Cellular senescence, a stable proliferative arrest in normal cells, is triggered by a combination of four major mechanisms: telomere shortening-dependent replicative senescence (RS), oncogene-induced senescence (OIS), tumor suppressor gene (TSG) inactivation, as well as stress-induced senescence (Ogrunc et al., 2014). RS, also known as replicative senescence often depends on telomere dysfunction induced by replication-associated telomere shortening (Petrova et al., 2016). OIS depends on activation or overexpression of oncogenes such as RAS and BRAF^V 600E^ activation (Xie et al., 2015). TSG depends on tumor suppressor inactivation or loss, such as PICS (Tan et al., 2015). During each type of senescence, various phenotypes were consequently altered such as senescence-associated β-galactosidase activity (SA-β-gal), formation of SAHF, and sustained DDR (Petrova et al., 2016). Subsequently, p53, p21^waf^, and p16 are activated while cyclin-dependent kinases including CDK4 and CDK6 are contrarily suppressed in senescent cells (Petrova et al., 2016). Meanwhile, some of epigenetic modifiers were also involved such as H3K9 histone methyltransferase and polycomb complexes including H3K36 demethylase and H3K27 demethylase (David, 2012). Compared to other senescence mechanisms, stress-induced senescence plays a comprehensive and predominant role in tumor initiation by widely altering genomic and epigenetic landscapes. ROS, one of the earliest responses toward oxidative stress, exerts various functions even with apparently opposite outcomes. Concretely speaking, intercellular ROS accumulation effectively stimulated cell hyperproliferation via activating mitogenic pathway, whereas at the meantime, ROS contrarily suppressed DNA replication though causing DDR-p53/p21^waf^ signaling (Ogrunc et al., 2014). Thus, given the controversial role of ROS-induced senescence in tumorigenesis, the mechanisms underlying still need extensively investigation. At the meantime, considering the vital role of ROS in tumor initiation, developing an effective ROS-dependent pro-senescence agent is also urgent need for clinical chemoprevention.

Until now, various pro-senescence agents were developed and some of them have been entered into clinical trials such as GRN163L, nutlin, and MIRA-1 (Acosta and Gil, 2012; Burchett et al., 2014). However, systemic toxicity and drug resistance greatly limited their clinical applications. SHK, a pure compound isolated from the herbal medicine *Lithospermum erythrorhizon*, exhibited anti-inflammatory, antitumor, and antiviral effectiveness with tolerable toxicity (Chen et al., 2002; Lu et al., 2011). Previous studies indicated that SHK could significantly inhibit lung carcinogenesis by stimulating apoptosis and arresting cell cycle (Lan et al., 2014). Meanwhile, SHK also effectively suppressed tumor metastasis via suppressing ERK1/2 signaling (Wang et al., 2013). Also, SHK shown to induce apoptosis, senescence and necrosis through up regulating p53 in A549 cells (Yeh et al., 2015). Furthermore, in gefitinib-resistance lung cancer cells, SHK was also reported to sensitize chemotherapeutic efficacy by inhibiting TrxR while activating EGFR proteasomal degradation (Li et al., 2017). However, whether SHK could trigger ROS generation in lung cancer cells is unknown, and whether SHK-induced senescence depends on ROS generation also remains unclear.

In our present study, the pro-senescence effects and mechanisms of SHK were determined in A549 and H1299 cells *in vitro* and xenograft models *in vivo*. We found that SHK significantly inhibited tumor proliferation via stimulating cellular senescence. Further mechanistic study revealed that SHK-induced senescence was dependent on ROS generation with increasing DNA damage (p-H2A.X), p53 (total and phosphorylation), and p21^waf^. Our study suggested that SHK, a ROS-dependent senescence inducer, could serve as a promising agent for further lung cancer treatment, and even chemoprevention.

## Materials and Methods

### Reagents and Cell Culture

SHK (purity > 98%) was purchased from Chengdu Must Bio-technology Co., Ltd. (Chengdu, China). MTT and NAC were purchased from Sigma-Aldrich (St. Louis, MO, United States). PI staining was purchased from Biosciences (BD Biosciences, Franklin Lakes, NJ, United States). β-actin, MEK-1, p53 and p21^waf^ primary antibodies and HRP-conjugated secondary antibody were purchased from Santa Cruz Biotechnologies Inc. (Santa Cruz, CA, United States). Bcl-2, Bax, Rb, Cleaved Caspase-3, Ras, H3K36me2, p-p53, p-H2A.X, Histone H3 and Cyclin D1 antibodies were purchased from Cell Signaling Technology Inc. (Beverly, MA, United States). Kdm2b antibody was purchased from Millipore (Burlington, MA, United States). p16 antibody was purchased from Abcam (Cambridge, United Kingdom). Specific small interfering RNA (siRNA) of p21 and riboFECTTM CP Transfection Kit (166T) were purchased from Ribobio, Co., Ltd (Guangdong, China). Two human LAC cells (A549 and H1299) were purchased from ATCC (Manassas, VA, United States). A549 and H1299 cells were maintained in RPMI-1640 medium with 10% fetal bovine serum (FBS) and 1% penicillin-streptomycin solution, and cultured in a humidified atmosphere at 37°C with 5% CO_2_.

### MTT Assay

Cells were seeded into 96-well plates at 2 × 10^3^ cells/well. After cultured overnight, cells were treated with SHK (0–6 μM) for 24, 48, and 72 h. Cells were incubated with the MTT solution (0.5 mg/mL) at 37°C for 4 h. The supernatants were replaced by dimethyl sulfoxide (150 μL/well). Then the absorbance was measured using microplate reader Victor X3 (PerkinElmer, Waltham, MA, United States) at 490 nm. The half inhibitory concentration (IC_50_) was obtained using the non-linear regression program of GraphPad Prism 5.

### 5-Ethynyl-2′-deoxyuridine (EdU) Assay

EdU, a nucleoside analog of thymidine which is in corporated into DNA during active DNA synthesis, is often used to measure cell proliferation. Cells were seeded into 96-well plates at 2 × 10^3^ cells/well, and treated with SHK (1 and 2 μM for A549, 0.3 and 0.6 μM for H1299, respectively) for 72 h. Then cells were incubated with EdU labeling medium (50 μM) for 2 h at 37°C with 5% CO_2_, fixed with 4% paraformaldehyde (pH = 7.4) for 30 min, and incubated with glycine for 5 min. After rinsed with PBS, cells were incubated with anti-EdU working solution at room temperature for 30 min, following with Hoechst 33342 dye (1×) at room temperature for 30 min. The images were captured using Leica3000B (Leica, GER). The percentages of EdU-positive cells were calculated from five random fields in two wells.

### Cell Cycle Analysis

Cells were seeded into 6-well plates at 6 × 10^4^ cells/well and treated with SHK (1 and 2 μM for A549, 0.3 and 0.6 μM for H1299, respectively) for 72 h. Following, cells were harvested, washed twice with PBS, and fixed with 70% cold ethanol at 4°C overnight, then stained with PI (50 μg/mL) at 37°C for 0.5 h protecting from light. Cell cycle was detected by flow cytometry (BD Biosciences, San Jose, CA, United States). The results were analyzed by FlowJo 7.6 software.

### Detection of Senescence-Associated Heterochromatin Foci (SAHF)

SAHF are specialized domains of facultative heterochromatinin senescent cells. 4′,6-diamidino-2-phenylindole (DAPI) staining was used to observe SAHF. Cells were seeded into 15 mm confocal dish at 3 × 10^4^ cells/well, cultured overnight, then treated with SHK (2 μM for A549 and 0.6 μM for H1299, respectively) for 72 h. After harvested, cells were fixed in 4% cold paraformaldehyde, incubated with DAPI for 10 min. The images were captured using Leica TCS SP8 confocal microscope (Leica, GER). All images were acquired using an identical parameter time for all samples.

### Senescence-Associated β-Galactosidase (SA-β-Gal) Assay

Cells were seeded into 6-well plates at 6 × 10^4^ cells/well and treated with SHK (1 and 2 μM for A549, 0.3 and 0.6 μM for H1299, respectively) for 72 h. Then cells were stained with SA-β-Gal in accordance to the manufacturer’s procedure of Senescent Cell Histochemical Staining Kit (CST). Meanwhile, tumor tissues were frozen and sliced up (4 μm). Then the frozen sections were stained immediately with SA-β-Gal in accordance to the manufacturer’s procedure of Senescent Cell Histochemical Staining Kit (CST).

### ROS Detection

Reactive oxygen species formation in cells was detected using Cellular Reactive Oxygen Species Detection Assay Kit (Abcam, United Kingdom), according to the manufacturer’s instruction. Briefly, cells were seeded into 6-well plates at 6 × 10^4^ cells/well and treated with SHK (1 and 2 μM for A549, 0.3 and 0.6 μM for H1299, respectively) for 72 h. Then the cells were collected and washed twice with PBS and suspended in PBS-H_2_DCFDA (10 μM) at 37°C for 30 min. After that, the fluorescence was monitored by flow cytometry.

### siRNA Interference

The siRNA was used to interfere the gene and protein expressions of p21^waf^. The sequence of siRNA was: GAATGAGAGGTTCCTAAGA. Cells were seeded into 6-well plates at 3 × 10^5^ cells/well, and transfected with 50 nM siRNA or negative control using riboFECTTM CP Transfection Kit (166T) in serum free Opti-MEM media according to the manufacturer’s protocal. After 12 h transection, the cells were digested and seeded into new 6-well plates at a density of 1 × 10^5^ cells/well, and cultured overnight, then the cells were continuously cultured with or without SHK (2 μM for A549 and 0.6 μM for H1299, respectively) for 72 h. Finally, cells were harvested for following assay.

### Western Blot

After cells were harvested, total proteins were extracted using RIPA lysis buffer and phenylmethanesulfonyl fluoride (PMSF), and quantified using Coomassie Brilliant Blue Kit (Bio-Rad, Hercules, CA, United States). Protein samples were separated by SDS-PAGE, transferred onto PVDF membranes, and blocked with 5% BSA for 1 h, and then incubated with the primary antibodies at 4°C overnight, subsequently, with secondary antibodies at room temperature for 1 h. ECL chemiluminescence reagent was applied to detect for fluorescent signals using FluorChem E (Santa Clara, CA, United States). Protein bands were quantified using Quantity One software (Bio-Rad, Hercules, CA, United States).

### Apoptosis Detection

Cells were seeded into 6-well plates at 6 × 10^4^ cells/well and treated with SHK (1 and 2 μM for A549, 0.3 and 0.6 μM for H1299, respectively) for 72 h. After harvested, cells were processed using the FITC annexin V/PI Apoptosis Detection Kit (BD Biosciences, United States), according to the manufacturer’s protocol. After that, cell apoptosis was analyzed by the flow cytometry.

### Immunofluorescence

Cells were seeded into 15 mm confocal dish 3 × 10^4^ cells/well, cultured overnight, then treated with SHK (1 and 2 μM for A549, 0.3 and 0.6 μM for H1299, respectively) for 72 h. After harvested, cells were fixed in 4% cold paraformaldehyde, incubated with the primary antibodies at 4°C overnight, and with Alexa Fluor^®^ conjugated secondary antibodies (1:500, v/v) at room temperature for 1 h protecting from light. The images were captured using Leica TCS SP8 confocal microscope (Leica, GER). All images were acquired using an identical parameter time for all samples.

### NSCLC Xenograft Mice Models

Female Balb/c-nude mice (4–6 weeks, 16–20 g) were purchased from Laboratory Animal Center of Sun Yat-sen University [Guangzhou, China; License number: SCXK (Guangdong) 2016-0029], and kept in the animal facility in the SPF animal laboratory [License number: SYXK (GZ) 2014-0144] at International Institute for Translational Chinese Medicine, Guangzhou University of Chinese Medicine (Guangzhou, China). The animal experiments were approved by the Guangzhou University of Chinese Medicine Animal Care and Use Committee (Guangzhou, China), and conducted according with the ethical standards and national guidelines. For A549 xenograft model, mice were subjected to subcutaneously injection with A549 cells (2.5 × 10^6^ cells/mouse) in each right flank. Tumor volume (TV), defined based on two dimensions (L, long diameter; W, wide diameter), was measured by calipers, and calculated as formula: TV (mm^3^) = (L × W^2^)/2. When the tumors reached a mean volume of 50 mm^3^, eligible mice were randomized into four groups (*n* = 7) as following: gavage control (sterilized coin oil), afatinib (5 mg/kg), SHK (5, 10 mg/kg). For H1299 xenograft model, mice were subjected to subcutaneously injection with H1299 cells (3 × 10^6^ cells/mouse) in each right flank. When the tumors reached a mean volume of 50 mm^3^, the mice were randomized into two groups (*n* = 7) as following: gavage control (sterilized coin oil), SHK (10 mg/kg). Animals received oral administration with drugs five times a week for 4 weeks. Body weights were recorded every 3 days. At the end of the experiments, mice were sacrificed, and the tumors and organs were removed and weighed for following assays.

### Immunohistochemistry

Tumor tissues were fixed in 4% paraformaldehyde, embedded in paraffin, and then sliced up (4 μm). Then slices were dewaxed, hydrated, and then incubated with natrium citric (0.01 M) for antigen retrieval. Following, the slices were rinsed with PBS, and incubated with diluted anti-p-H2A.X, anti-p53 and anti-p21 overnight at 4°C. Following steps were performed using the immunostaining kit (BOSTER Biological Technology) based on the manufacturer’s instructions.

### Statistical Analysis

All data were expressed as the mean ± standard deviation (SD). Significant differences were analyzed by one-way analysis of variance (ANOVA) followed by the LSD *post hoc* test using SPSS 19.0 software. Statistical differences were considered significant at *p* < 0.05.

## Results

### SHK Suppressed Lung Cancer Cell Proliferation via Arresting Cell Cycle at G0/G1 Phase

**FIGURE 1:**
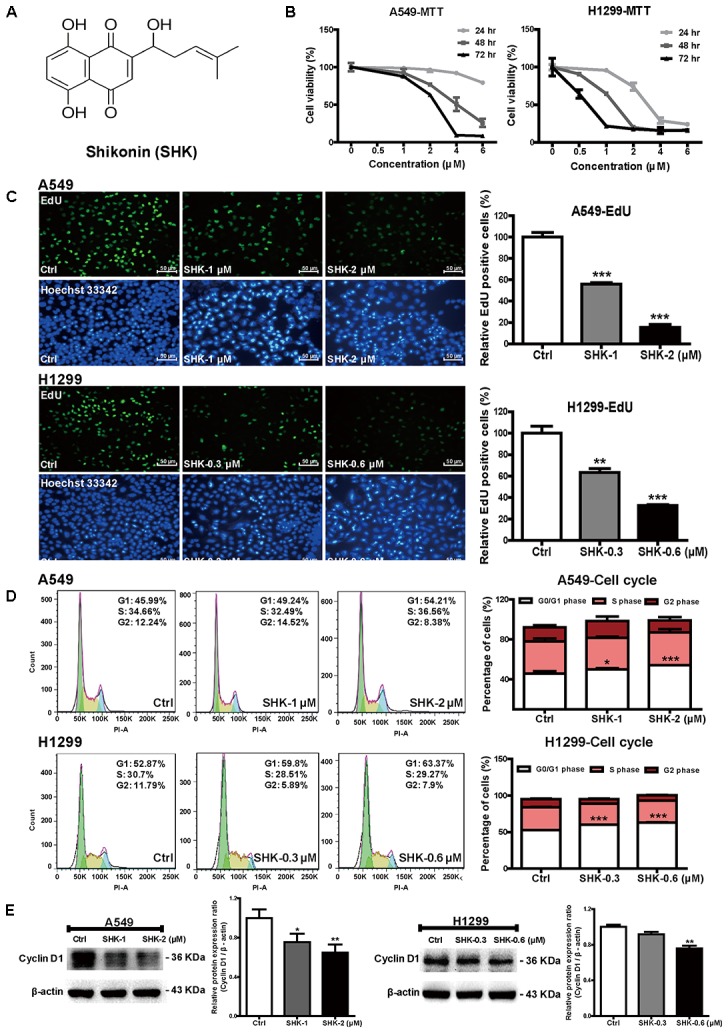
Effects of SHK on cell proliferation and cell cycle arrest. **(A)** Chemical structure of SHK (MW = 288.3). **(B)** Cell viabilities of A549 and H1299 cells were measured by MTT assay after treatment with SHK (0–6 μM) for 24, 48, and 72 h. **(C)** EdU incorporation was evaluated after SHK treatments for 72 h, and mean EdU fluorescences in SHK treatments were relative to that in control treatment, which was arbitrarily set as 100. **(D)** Percentages of cell cycle arrest were analyzed by flow cytometry after SHK treatments. **(E)** Expression of Cyclin D1 in A549 and H1299 cells after SHK treatments. Data represent mean ± SD (*n* = 3). ^∗^*P* < 0.05, ^∗∗^*P* < 0.01, ^∗∗∗^*P* < 0.001 *vs*. Ctrl (control).

To determine the cytotoxicity of SHK (**Figure 1A**), cell viabilities were evaluated at 24, 48, and 72 h, respectively by MTT assay. SHK dose- and time-dependently suppressed viable cell percentages in A549 and H1299 cells, and the respective IC_50_ were 2.28 ± 0.19 and 0.64 ± 0.07 μM at 72 h (**Figure 1B**). Therefore, considering diverse SHK sensitivity observed in these two cells, 1 and 2 μM of SHK were selected for further treatments in A549 cells, while 0.3 and 0.6 μM were applied to H1299 cells according to the equivalent viability in A549 cells.

Furthermore, EdU assay and flow cytometry were performed to analyze the effect of SHK on lung cancer cell proliferation. Given the cytotoxicity of SHK found previously (**Figure 1B**), the average percentages of EdU fluorescence, rather than the absolute EdU-labeling cell numbers, in each treatment were analyzed accordingly (Yang et al., 2011). In contrast to the abundant EdU-positive cells observed in control (Ctrl, 0.1% DMSO, same as follows), SHK at 1 and 2 μM significantly suppressed A549 cell proliferation ratio by 44.00% ± 2.22% and 84.42% ± 4.57% (**Figure 1C**, *P* < 0.001). Simultaneously, SHK at 2 μM notably increased the percentage of A549 cells at G0/G1 phase from 45.79% ± 2.27% in control treatment to 54.12% ± 0.10% (**Figure 1D**, *P* < 0.001). Similarly, remarkable inhibition of EdU fluorescence (range 36.51–67.44%) were also found in H1299 cells after treatment with 0.3 and 0.6 μM of SHK (**Figure 1C**, *P* < 0.001). And 0.3 and 0.6 μM of SHK also markedly arrested H1299 cells at G0/G1 phase by 13.78% ± 0.58% and 19.81% ± 0.47% (**Figure 1D**, *P* < 0.001). Moreover, given the significant G0/G1 arrest in A549 and H1299 cells, cyclin D1, a key protein required for progression through the G1 phase of the cell cycle (Sherr and Roberts, 2004) was further determined. Compared to control treatment, SHK at 2 and 0.6 μM significantly decreased the expression of cyclin D1 by 35.21% ± 8.17% and 24.25% ± 5.73% in A549 and H1299 cells, respectively (**Figure 1E**, *P* < 0.01).

### SHK Notably Promoted Cell Senescence in A549 and H1299 Cells

Considering two key phenotypes of senescence, G0/G1 arrest (Lleonart et al., 2009) (**Figure 1D**) and cell relative enlargement (Muñoz-Espín and Serrano, 2014) (**Figure 1C**), were unexpectedly observed after our SHK treatments, we hypothesized that SHK might influence senescence to suppress cell proliferative capacity in A549 and H1299 cells. Thus, the effects of SHK in senescence were further determined by SAHF detection (Funayama et al., 2006) and SA-β-Gal staining (Dimri et al., 1995).

**FIGURE 2:**
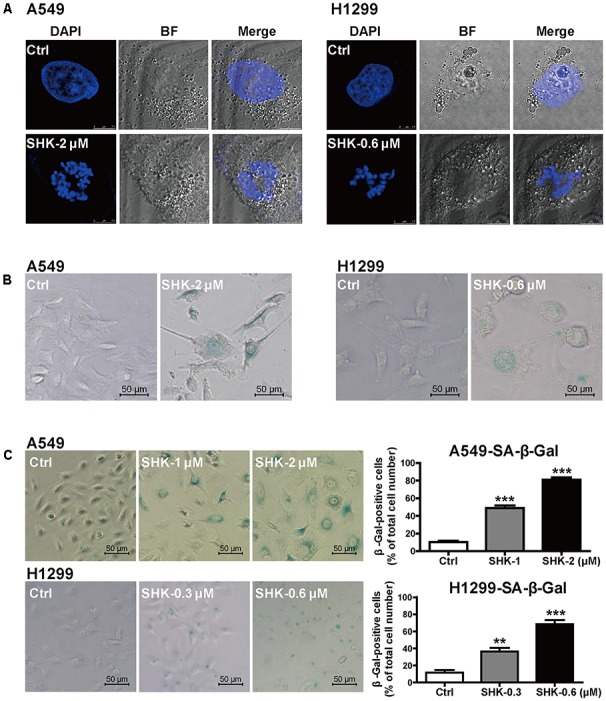
SHK induced senescence in A549 and H1299 cells. **(A)** Representative confocal imaging (scale bar: 7.5 μm) of SAHF in A549 and H1299 cells after SHK treatment using DAPI (blue) staining. **(B)** Morphologic changes of A549 and H1299 cells were observed after SHK treatment. **(C)** The proportions of senescence A549 and H1299 cells were determined using SA-β-Gal staining after SHK treatments. ^∗∗^*P* < 0.01, ^∗∗∗^*P* < 0.001 *vs*. Ctrl (control).

SAHF, specialized domains of facultative heterochromatin, are predominately contributed to cellular senescence through repressing proliferative-promoting proteins such as cyclin A and D (Banumathy et al., 2009; Litwiniec et al., 2013). To elucidate mechanism underlying the SHK-induced cell cycle arrest observed in **Figure 1D**, SAHF was detected by confocal microscope. Since most of cellular morphological observation was not closely dependent on dosage, only relative high doses of SHK (2 and 0.6 μM respectively in A549 and H1299 cells) were used. After treatment with SHK, the chromosomes in control were markedly condensed into numerous SAHF focus. In addition, compared to H1299 cells, SAHF focus was abundantly condensed in A549 cells, suggesting A549 was more sensitive for SHK-induced senescence (**Figure 2A**). Furthermore, both in A549 and H1299 cells, SHK remarkably altered cellular morphology from spindle and round to flat and enlarged, and the presence of vacuoles were also observed, especially in A549 cells (**Figures 2B,C**). Meanwhile, the proportions of senescent cells, positively labeled by β-Gal (Chen et al., 2015), were also gradually increased by SHK (**Figure 2C**), either in A549 cells (induction ranging from 3.77- to 6.89-fold) (*P* < 0.001) or in H1299 cells (induction ranging from 2.10- to 4.83-fold) (*P* < 0.01 or *P* < 0.001).

### SHK Induced Cellular Senescence Through Stimulating ROS Generation and Subsequently Triggering DNA Damage-p53/p21^waf^ Axis

**FIGURE 3:**
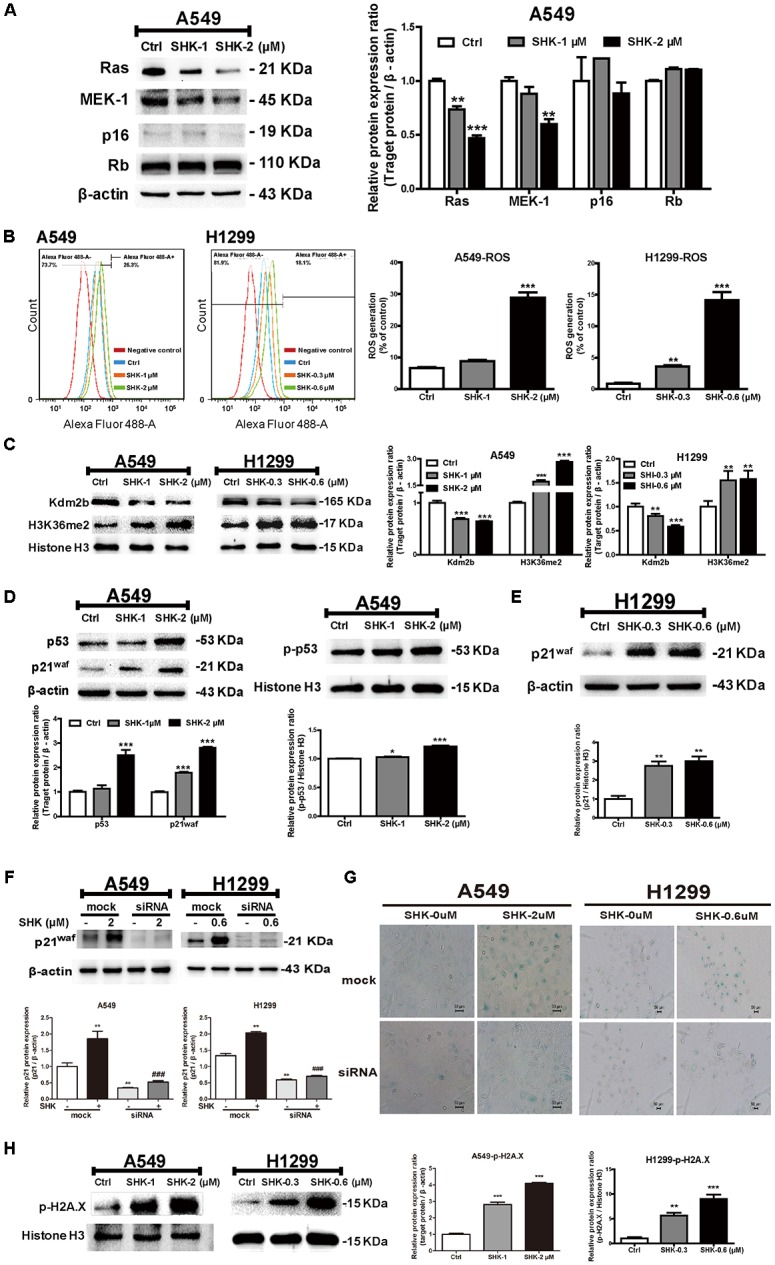
The underlying mechanism of SHK-induced senescence in A549 and H1299 cells. **(A)** Expressions of Ras, MEK-1, p16 and Rb in A549 cells after SHK treatments. **(B)** Levels of ROS generation in A549 and H1299 cells were detected by flow cytometry after SHK treatments. **(C)** Expressions of Kdm2b and H3K36me2 were measured by Western blot after SHK treatments. **(D)** Expressions of p53 and p21^waf^ in total protein, and p-p53 in nuclei were determined by Western blot after SHK treatments. **(E)** Expressions of p21^waf^ in H1299 cells were measured by Western blot after SHK treatments. **(F)** Expressions of p21^waf^ in A549 and H1299 cells transfected with siRNA after SHK treatments. **(G)** Representative SA-β-Gal staining imaging (scale bar: 50 μm) of A549 and H1299 cells transfected with siRNA after SHK treatments. **(H)** Expression of p-H2A.X was inspected by Western blot after SHK treatments. ^∗^*P* < 0.05, ^∗∗^*P* < 0.01, ^∗∗∗^*P* < 0.001 *vs*. Ctrl (control), ^###^*P* < 0.001 *vs*. SHK.

Cellular senescence is predominantly triggered via either oncogenes activation (RAS and BRAF^V 600E^) or depletion of tumor suppressor genes [TSG: PTEN, RB, and CDKN2A (encoding p16 protein)] (Nardella et al., 2011). Herein, to further determine the mechanism underlying SHK-induced cell senescence, Ras, MEK-1, Rb, and p16 expressions were evaluated only in A549 cells due to the abnormal basal expressions of oncogenes and TSG in p53-deleted H1299 cells. Rather than activation, Ras and MEK-1 expressions were unexpectedly decreased by SHK (**Figure 3A**, *P* < 0.05), meanwhile, no significant differences were observed on Rb and p16 expressions (**Figure 3A**), suggesting that oncogene and TSG maybe not the main driver factors in SHK-induced senescence.

On the contrary, ROS production was notably increased by SHK (ranging from 0.32- to 3.36-fold in A549 cells and 3.15- to 15.31-fold in H1299 cells) (**Figure 3B**, *P* < 0.01). Meanwhile, Kdm2b/JHDM1b, an H3K36me2-specific demethylase involved in cellular senescence, was reported to effectively suppress ROS generation (Polytarchou et al., 2008). After SHK treatment, we found that the expression of Kdm2b was significantly decreased in A549 and H1299 cells (**Figure 3C**, *P* < 0.01). In contrast, the protein level of H3K36me2 was dramatically increased with remarkable inductions (**Figure 3C**, *P* < 0.01).

Previous study revealed that ROS contributed to cell senescence through the activation of the p53/p21^waf^ pathway (Adams, 2009). Thus, to further determine the mechanism of SHK-induced senescence and ROS generation, p53 and its downstream target p21^waf^ were evaluated. As shown in **Figure 3D**, in A549 cells, SHK effectively stimulated p53 transcription, and subsequently induced p21^waf^ expression (*P* < 0.001). Meanwhile, the phosphorylated p53 in nucleus was significantly increased by SHK (**Figure 3D**, *P* < 0.01). Whereas, in p53-deleted H1299 cells, the presence of p21^waf^ could alternatively lead to growth arrest and differentiation in a p53-independent manner (Wang et al., 1999). Accordingly, we also observed that SHK at 0.3 and 0.6 μM could markedly induced p21^waf^ protein level by 1.76- and 2.00-fold, respectively (**Figure 3E**, *P* < 0.01). To further confirm the role of p21^waf^ in SHK-induced senescence, p21^waf^ was knock-down in A549 and H1299 cells. The mRNA and protein expressions of p21^waf^ in A549 and H1299 cells were obviously down-regulated by siRNA (Supplementary Figure S1). After siRNA interference, p21^waf^ expression was remarkably reduced by 71.90% and 65.52%, respectively, compared to that in SHK treated cells (**Figure 3F**). Moreover, siRNA interference also significantly reversed SHK-induced senescence in this two cell lines (**Figure 3G**).

Previous study suggested that after senescence induction, the activation of p53/p21^waf^ pathway was triggered by sever DDR (Dolan et al., 2015). Compared to control treatment, SHK at 1 and 2 μM enhanced the expressions of p-H2A.X, sensor for DNA damage, by 1.97- and 3.47-fold, respectively in A549 cells; while that were also increased by 0.3 and 0.6 μM of SHK with respective induction of 4.68- and 8.03-fold in H1299 cells (**Figure 3H**, *P* < 0.01).

### SHK-Induced Senescence Was Specifically Dependent on ROS Generation

**FIGURE 4:**
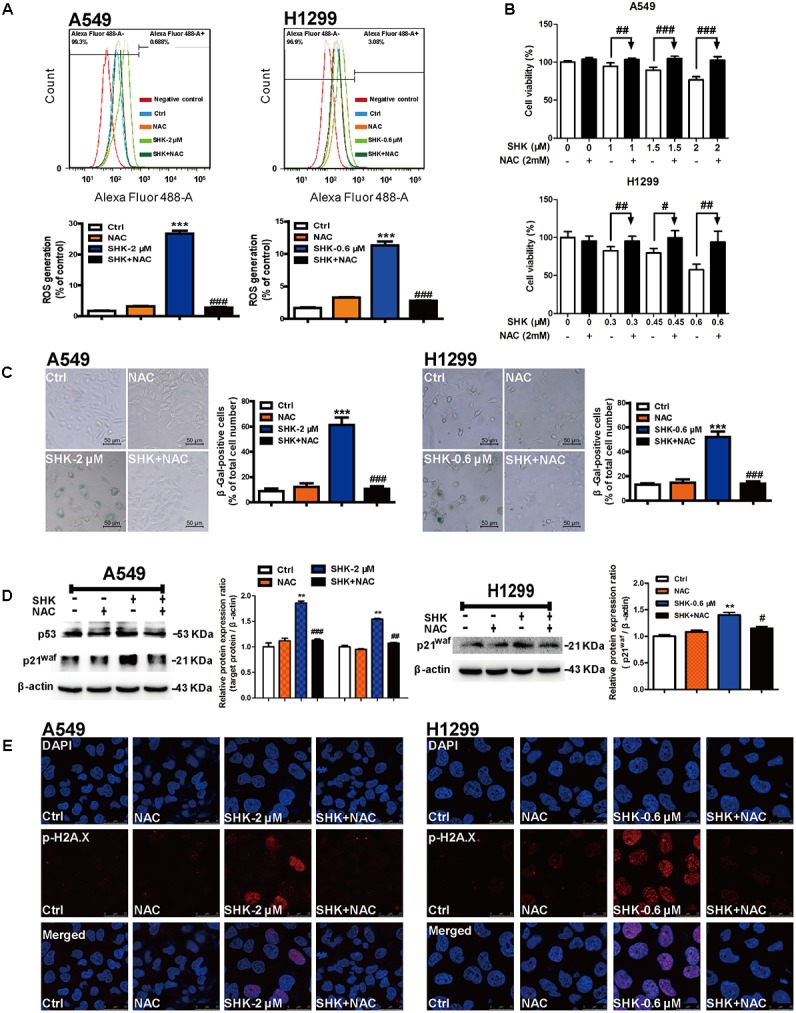
SHK-induced senescence is specifically dependent on ROS generation. **(A)** ROS generations in A549 and H1299 cells were detected by flow cytometry after 1 h of NAC (2 mM) pretreatment and 72 h of SHK treatment. **(B)** Cell viabilities of A549 and H1299 were determined in the presence of 2 mM NAC pretreatment for 1 h. **(C)** The proportions of senescence A549 and H1299 cells were determined using SA-β-Gal staining after NAC pretreatment and SHK treatment. **(D)** Expressions of p53 and p21^waf^ in A549 cells, and expression of p21^waf^ in H1299 cells were detected by Western blot after NAC pretreatment and SHK treatment. **(E)** Representative confocal imaging (scale bar: 25 μm) of double-stained cells after NAC pretreatment and SHK treatment using p-H2A.X (red) and DAPI (blue) staining. ^∗∗^*P* < 0.01, ^∗∗∗^*P* < 0.001 *vs*. Ctrl (control), ^#^*P* < 0.05, ^##^*P* < 0.01, ^###^*P* < 0.001 *vs*. SHK.

To evaluate whether SHK-induced senescence is dependent on ROS, NAC, a classic ROS scavenger was used in our further study. Compared to low intercellular ROS level in control treatment, no significant alteration was observed after treatment with NAC (2 mM). While, NAC pretreatment notably attenuated SHK-induced intracellular ROS generation by 89.55% ± 0.99% and 75.52% ± 1.35% in A549 and H1299 cells, respectively (**Figure 4A**, *P* < 0.001).

In contrast to gradually suppressed cell viability in SHK treatments, pretreated with NAC successfully reversed the cytotoxicity of SHK in A549 and H1299 cells in dose-dependent manner (**Figure 4B**). At the same time, SA-β-Gal staining results also showed that NAC pretreatment markedly reduced the percentages of SHK-induced senescent cells by 82.64% ± 5.68% and 73.47% ± 6.63% in A549 and H1299 cells, respectively (**Figure 4C**, *P* < 0.001). And the morphologies of SHK-induced enlarged and flat senescent cells were also effectively reversed by NAC to spindle and round (**Figure 4C**). Moreover, SHK-induced p53 and p21^waf^ activation was also completely reversed by pretreated with NAC in A549 cells, as well as that of p21^waf^ activation in H1299 cells (**Figure 4D**). Additionally, immunofluorescence results also revealed that both in A549 and H1299 cells, SHK-induced p-H2A.X over-expressions were dramatically diminished by NAC pretreatment (**Figure 4E**).

### SHK-Induced Apoptosis Was Dependent on ROS Generation

**FIGURE 5:**
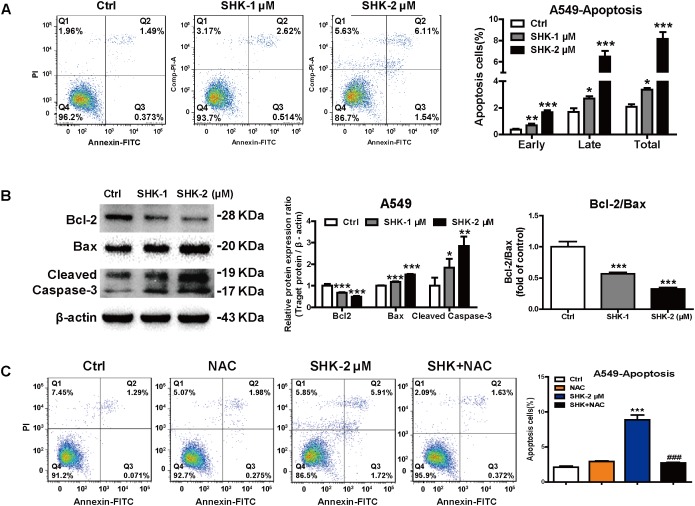
SHK-induced apoptosis depending on ROS generation. **(A)** The proportions of apoptosis cells of A549 were determined by flow cytometry using FITC annexin V/PI double staining assay after SHK treatments. **(B)** Expressions of Bcl-2, Bax and Cleaved Caspase-3 were measured by Western blot after SHK treatments. **(C)** The proportions of apoptosis cells of A549 were determined by flow cytometry after NAC pretreatment and SHK treatment using FITC annexin V/PI double staining assay. ^∗^*P* < 0.05, ^∗∗^*P* < 0.01, ^∗∗∗^*P* < 0.001 *vs*. Ctrl (control), ^###^*P* < 0.001 *vs*. SHK.

Emerging evidence demonstrated that cytotoxic chemotherapeutic agents simultaneously stimulated a robust cellular senescence to achieve “synthetic senescent interaction,” but the extent of senescence and apoptosis is unknown (Collado et al., 2007). Meanwhile, previous study also revealed that apoptosis is the ultimate biological consequence for senescent cells *in vivo* (Baker et al., 2011). Thus, apoptosis ratio was analyzed subsequently in A549 cells, which were more sensitive toward SHK-induced senescence (**Figures 2A–C**). Compared to control treatment, SHK significantly increased early and late phases of apoptosis, especially SHK at 2 μM induced that by 4.55- and 3.96-fold, respectively (**Figure 5A**, *P* < 0.001). However, compared to the significantly stimulated senescence in A549 cell (range 38.70%–70.69%, **Figure 2C**), the percentage of total apoptosis is extremely low (range 3.13%–7.65%, **Figure 5A**). These implied that rather than apoptosis, the predominant effect of SHK on LAC cells was stimulating senescence.

Moreover, western blot results also showed that compared with control treatment, SHK markedly decreased the expression of anti-apoptosis protein Bcl-2, while that of pro-apoptotic Bax and Cleaved Caspase-3 were significantly increased. Meanwhile, SHK simultaneously decreased Bcl-2/Bax ratio, a parameter determined the susceptibility of cellular apoptosis, by 43.35% ± 2.60% and 67.74% ± 2.42%, respectively (**Figure 5B**, *P* < 0.001). Moreover, NAC pretreatment significantly diminished SHK-induced apoptosis from 8.87% ± 1.17% to 2.70% ± 0.17% in A549 cells (**Figure 5C**, *P* < 0.001).

### SHK Suppressed Lung Cancer Growth in NSCLC Xenograft Mice Models

**FIGURE 6:**
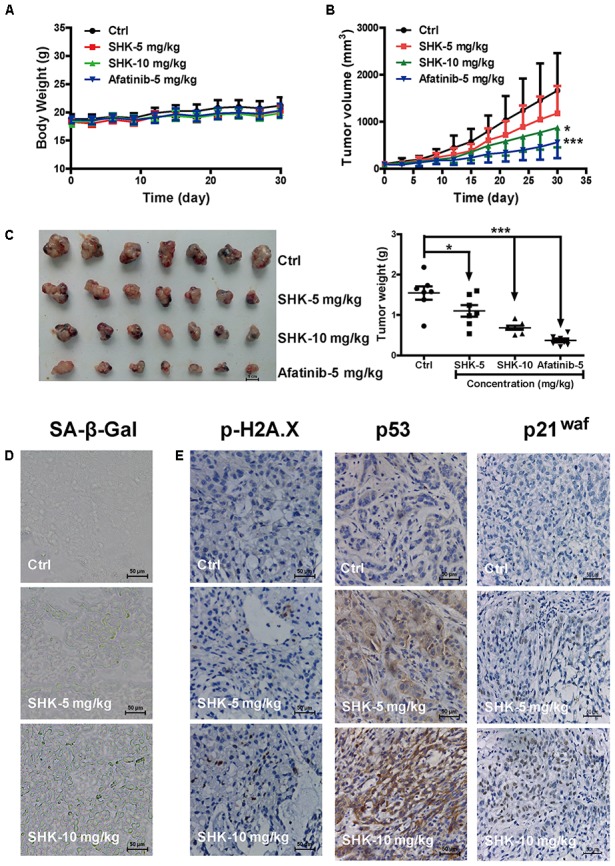
Inhibitory effects of SHK in A549 xenograft mice model. **(A)** Average body weight curves of Balb/c-nude mice (*n* = 7). **(B)** Tumor volume ((L × W^2^)/2, L, long diameter; W, wide diameter) were analyzed. **(C)** Tumors were photographed and tumor weights were analyzed. **(D)** Images of senescence cells in tumor consecutive frozen sections using SA-β-Gal staining assay (scale bar: 50 μm). **(E)** Images of tumor p-H2A.X, p53 and p21^waf^ protein expressions were presented by immunohistochemistry (scale bar: 50 μm). ^∗^*P* < 0.05, ^∗∗∗^*P* < 0.001 *vs*. Ctrl (control).

To further determine the anti-tumor effect of SHK-induced senescence, A549 xenograft model was established. Afatinib, a tyrosine kinase inhibitor normally used as a first-line chemotherapeutic agent for LAC, was reported to induce irreversible senescence to substantially suppress tumor progression (Mcdermott et al., 2016). Given SHK is supposed to be a good senescence-targeting agent in our study, afatinib was used as positive control. After orally gavage SHK and afatinib for 4 weeks, no significant alterations on average body weight were observed among treatments (**Figure 6A**). Meanwhile, compared to control treatment (Corn oil), SHK, either at 5 mg/kg or at 10 mg/kg, did not remarkably alter the tissue indexes including heart, liver, spleen, lung, and kidney (Supplementary Figure S2A), suggesting no systemic toxicity was observed after SHK treatments.

Notably, SHK dose-dependently suppressed tumor growth (**Figure 6B**), tumor size and tumor weight (**Figure 6C**). Meanwhile, compared to control, SHK at 5 mg/kg and 10 mg/kg also significantly decreased the tumor weights by 28.57% and 55.84%, respectively (**Figure 6C** and **Table 1**, *P* < 0.05).

**Table 1:** The inhibitory effects of SHK on A549 xenograft mice.

Groups	Dose (mg/kg)	Routes	Numbers	Body weight (g)	Tumor weight (g)	Inhibitory rate (%)
Control	Corn oil	p.o.(qd)	7/7	21.2 ± 1.46	1.54 ± 0.44	
SHK	5	p.o.(qd)	7/7	20.3 ± 0.8	1.10 ± 0.38	28.57
SHK	10	p.o.(qd)	7/7	19.9 ± 0.86	0.68 ± 0.14	55.84
Afatinib	5	p.o.(qd)	7/7	20.3 + 1.22	0.37 ± 0.13	75.97

To evaluate whether the anti-tumor effect of SHK ascribe to senescence induction, cellular senescence, and the expressions of DNA damage sensor p-H2A.X, p53 and p21^waf^ were detected in tumor tissues. Compared to control treatment, SHK treatments dramatically increased the percentages of β-Gal-positive senescent cells in tumor tissues (**Figure 6D**). In addition, the expressions of p-H2A.X, p53 and p21^waf^ were also remarkably increased by SHK in a dose-dependent manner (**Figure 6E**).

**FIGURE 7:**
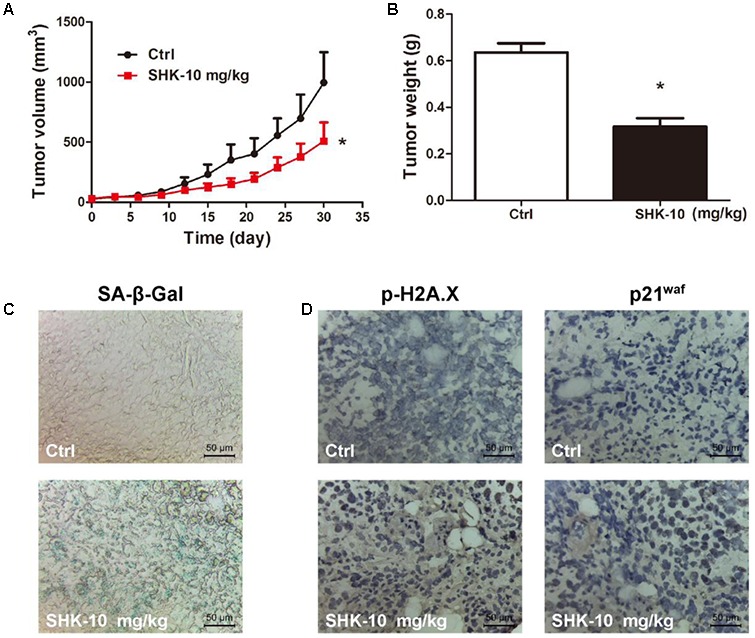
Anti-tumor effects of SHK in H1299 xenograft mice model. **(A)** Tumor volumes were analyzed. **(B)** Tumor weights were analyzed. **(C)** Images of senescence cells in tumor consecutive frozen sections using SA-β-Gal staining assay (scale bar: 50 μm). **(D)** Images of tumor p-H2A.X and p21^waf^ protein expressions were presented by immunohistochemistry (scale bar: 50 μm). ^∗^*P* < 0.05 *vs*. Ctrl (control).

Since p53-deleted H1299 cells is more sensitive than A549 cells to SHK (**Figure 1B**), we also evaluate the anti-tumor effects of SHK *in vivo* using H1299 xenograft model. Similar to the results in A549 xenograft model, SHK could effectively suppress tumor volume and tumor weight in H1299 xenograft mice model (**Figures 7A,B**). Compared to control treatment, 10 mg/kg of SHK remarkably inhibited tumor weights by 50.98%. Meanwhile, SHK treatment markedly increased the percentages of β-Gal-positive senescent cells in tumor tissues (**Figure 7C**). In addition, the expressions of p-H2A.X and p21^waf^ were also remarkably increased by SHK treatment (**Figure 7D**). No changes of the organ (heart, liver, spleen, lung, and kidney) indexes were found in all mice (Supplementary Figure S2B). H&E staining results also showed that no pathologic changes were observed in organs (heart, liver, and kidney) (Supplementary Figure S2C). These results suggested SHK could suppress lung tumorigenesis through stimulating cellular senescence.

## Discussion

In our study, the anti-tumor effect of SHK was determined and the senescence-involved mechanism was subsequently evaluated. We found that rather than inducing apoptosis, SHK predominantly stimulated cellular senescence via triggering DNA damage-p53/p21^waf^ axis. Moreover, SHK-induced senescence is closely dependent on intercellular ROS accumulation without activating oncogenes or suppressing TSG. Previous study demonstrated that cellular senescence activation could result in apparently opposite outcomes with either tumor suppressive or promoted effects in tumor initiation and progression (Rodier and Campisi, 2011). While the pro-senescence effect of SHK (10 mg/kg) led to strong tumor inhibition *in vivo* (55.84% in A549 xenograft mice, and 50.98% in H1299 xenograft mice, respectively), meanwhile, cell cycle, EdU, and apoptosis assays also revealed that SHK notably arrested cell proliferation and activated cellular apoptosis simultaneously. Wild-type p53 is a sequence-specific transcription factor that activates the transcription of downstream effector genes such as p21 et al., thereby preventing the transition from the G1 to the S phase of cell cycle (Slebos et al., 1994). In our study, besides of stimulating the expression and phosphorylation of p53, SHK also notably increased the percentage of A549 and H1299 cells at G0/G1 phase.

Our study provides additional evidence to complement the correlation between ROS and senescence, and suggested the district mechanism underlying ROS-induced senescence and OIS. Previous study demonstrated that ROS-induced senescence is partially triggered by OIS, intercellular ROS accumulation is ascribe to activation of RAS-RAF-MEK-ERK pathway (Muñoz-Espín and Serrano, 2014). On the contrary, we found that SHK significantly induced cellular senescence by accelerating ROS generation (**Figure 2C**) while inactivating oncogenes such as Ras and MEK-1 (**Figure 3A**), and meanwhile did not altering TSG such as RB (**Figure 3A**). This may suggest that ROS-induced senescence might be a combination progress of OIS and RS, and oncogene or TSG alterations are not essential driven factors. When ROS scavenger NAC is present, SHK-induced senescence were strongly diminished (**Figure 4C**), implied that SHK-induced senescence is closely dependent on ROS production than other molecular factors. Additionally, in a good agreement with another study, the negative correlation between ROS and oncogene expressions observed in our study suggested SHK may effectively reversed the over-activated oncogenes in tumors by physiologically enhanced ROS generation (DeNicola et al., 2011). Furthermore, ROS-induced senescence consequently induced DDR, p53, phosphorylated p53, and p21^waf^ (**Figures 3D,F**), and when ROS is depleted, DDR was subsequently suppressed (**Figure 4E**). Even in p53-depleted H1299 cells, senescence was also effectively stimulated by SHK through basal overexpressed p21^waf^ (**Figure 3E**). Moreover, knock-down of p21^waf^ significantly reversed SHK-induced senescence in both A549 and H1299 cells (**Figures 3F,G**), suggesting despite p53 phosphorylation (Nardella et al., 2011), persistent p21^waf^ activation is the most predominant prerequisite for ROS-induced senescence. Taken previous reports and our findings together may inform the further study on ROS-induced senescence in following aspects: (i) ROS-dependent senescence, not relay on oncogenes activation, is partially district from OIS, some alternative mechanism may underlying and need extensively investigation; (ii) p53 activation and phosphorylation could promote ROS-induced senescence, while p21^waf^ overexpression is the predominant driver.

Our findings uncovered the proportion and predominant role of senescence and apoptosis involved in anti-tumor effects of SHK. Although the concept of “senescence” is firstly found in 1965 by Leonard Hayflick, the usage of it as a cancer therapy strategy is underestimated and little excited pro-senescence agents were discovered (Petrova et al., 2016). This may partially due to the concerns of whether the senescent cells will relay on anti-apoptosis pathway to persist in tissues? And how the senescent cells will be cleaned up? We found that SHK significantly induced senescence and apoptosis at the same time (**Figures 2**, **5**). While compared to the abundantly stimulated senescence in A549 cell (range 38.70%–70.69%, **Figure 2C**), the percentage of total apoptosis is extremely low (range 3.13%–7.65%, **Figure 5A**), which implied that rather than apoptosis, the predominant mechanism responsible for the anti-tumor effect of SHK was ascribed to senescence stimulation. Meanwhile, SHK markedly decreased anti-apoptosis protein Bcl-2 while increased pro-apoptotic Bax (**Figure 5B**), suggested that SHK-induced senescent cells could be further and gradually cleaned up by SHK-induced apoptosis. Other studies also revealed that senescence stimulate immunosurveillance to eliminate tumor cells (Coppé et al., 2009; Rakhra et al., 2010), therefore, the effects of SHK on immune cells such as natural killer cells, T, B, and macrophages need to be further determined. Furthermore, compared to cytotoxic chemotherapeutic agents such as doxorubicin, vincristine, and cyclophosphamide, the pro-senescence agents could provide an alternative opportunity to avoid systemic toxicities by stimulating intrinsic anti-tumor response (Venturelli et al., 2013). In xenograft model, we also observed that SHK-induced senescence remarkably suppressed tumor growth without altering body weight during 4 weeks of treatment (**Figures 6A**). Moreover, No changes of the organ (heart, liver, spleen, lung, and kidney) indexes were found in all mice, and no pathologic changes were observed in organs (heart, liver, and kidney) (Supplementary Figure S2), suggesting no observed systemic toxicities were induced by SHK.

Taken together, by determining the pro-senescence mechanism of SHK in lung cancer cells, our present study advances our understanding of ROS-induced senescence and the anti-tumor effect of SHK in following aspects: (i) ROS-dependent senescence, not relay on oncogenes activation, is partially district from OIS, which implied that some alternative mechanisms may underlying and need extensively investigation; (ii) p53 activation and phosphorylation could promote ROS-induced senescence, while p21^waf^ overexpression might be the predominant driver; (iii) SHK may predominantly stimulate ROS-dependent senescence, while simultaneously accelerate cellular apoptosis program to eliminate the senescent tumor cells. The subsequently triggered pro-apoptosis pathway laid foundation to the further regard SHK as promising anti-tumor agents. However, considering the low proportion of apoptosis induced by SHK, other cleaned up mechanisms such as immunosurveillance, autophagy, and necrosis need to be further determined on SHK-induced senescence.

## Conclusion

SHK could induce cellular senescence through stimulating ROS generation and subsequently triggering DNA damage-p53/p21^waf^ axis. SHK, a ROS-dependent senescence inducer, could serve as a promising agent for further lung cancer treatment.

## Author Contributions

LL and ZL designed the research. HZ, QH, and ScH performed the study. LL, HZ, QH, and ScH analyzed the data, wrote and revised the manuscript. XY, TZ, WW, HW, SgH, LJ, YW, and XQ provided some technical support. All the authors are accountable for the content of the work.

## Conflict of Interest Statement

The authors declare that the research was conducted in the absence of any commercial or financial relationships that could be construed as a potential conflict of interest. The authors LL and ZL declared a shared secondary affiliation, though no collaboration with the handling Editor and reviewer WH. The reviewer WH and handling Editor declared their shared affiliation.

## Abbreviations

DDR: DNA damage response
EdU: 5-ethynyl-2′-deoxyuridine
LAC: Lung adenocarcinoma
MTT: 3-(4,5-dimethylthiazol-2-yl)-2,5-diphenyltetrazolium bromide
NAC: *N*-Acetyl-L-Cysteine
OIS: oncogene-induced senescence
PI: propidium iodide
PICS: PTEN loss-induced cellular senescence
PMSF: phenylmethanesulfonyl fluoride
ROS: reactive oxygen species
RS: replicative senescence
SA-β-Gal: senescence-associated β-galactosidase
SAHF: senescence-associated heterochromatin foci
SHK: shikonin
TSG: tumor suppressor genes
