# Supplementary material for: Senescence Inducer Shikonin ROS-Dependently Suppressed Lung Cancer Progression
Source: Front Pharmacol. 2018 May 23;9:519. doi: 10.3389/fphar.2018.00519 (PMC5974149; doi:10.3389/fphar.2018.00519)
Supplement: Supplementary file 1 [file Presentation_1.PDF]

Supplement Figure legend

Figure S1. mRNA (A) and protein (B) expressions of $p21^{waf}$ in A549 and H1299 cells transfected with siRNA.

Figure S2. Organ indexes in A549 (A) and H1299 (B) xenograft mice, represented as ratio of organ weight to body weight. (C) Representative H&E staining images of organs (heart, liver and kidney) in H1299 xenograft mice.

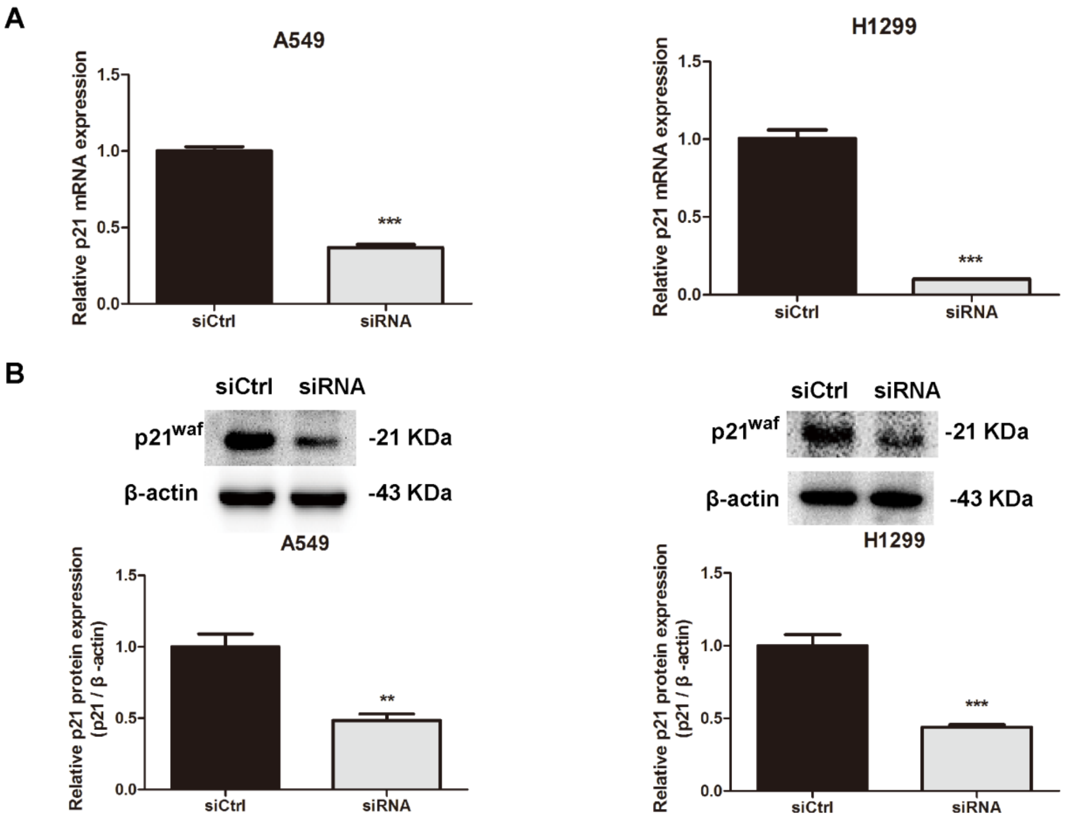

Supplement figure 1

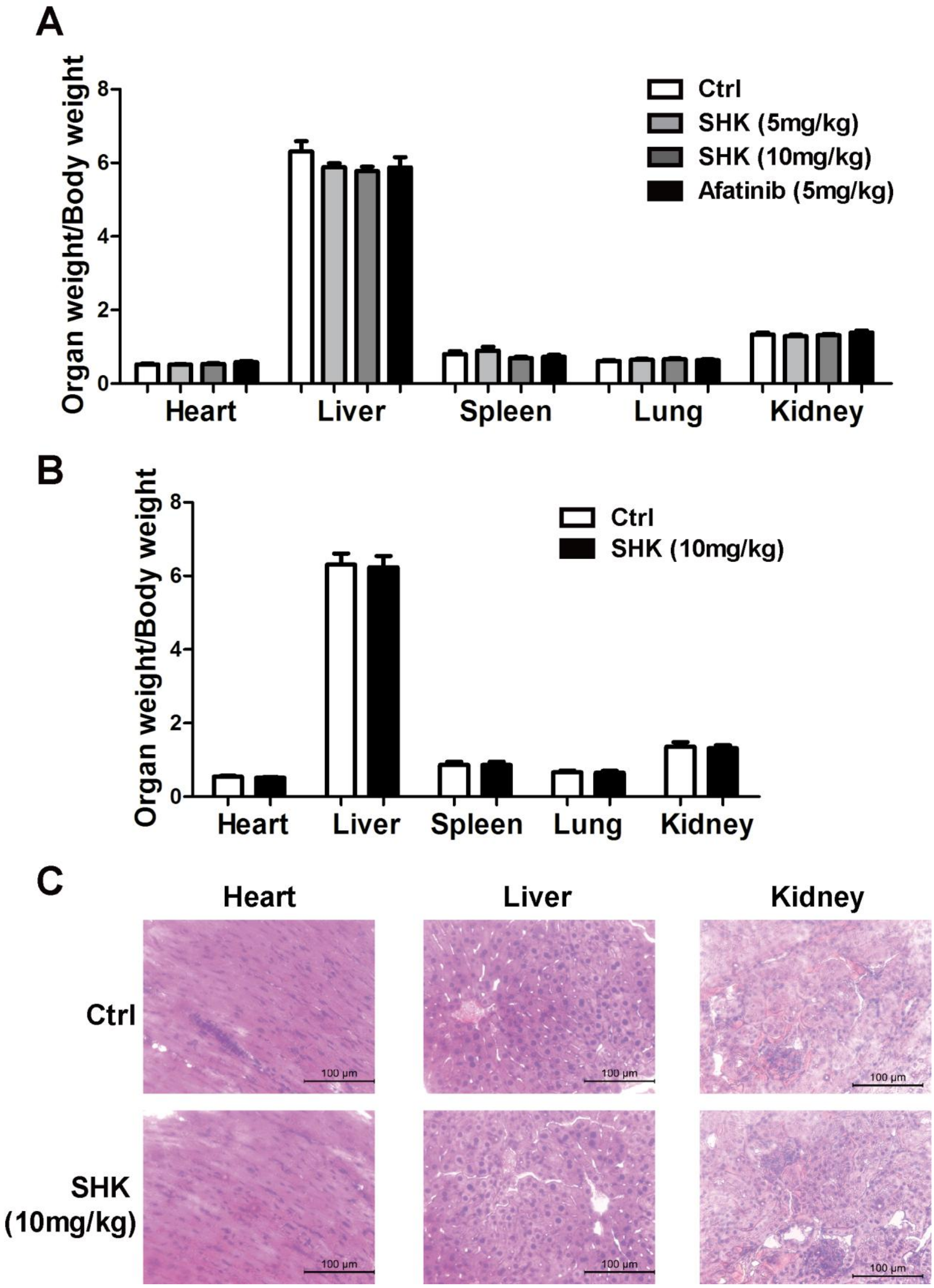

Supplement figure 2

Original data of Western blotting

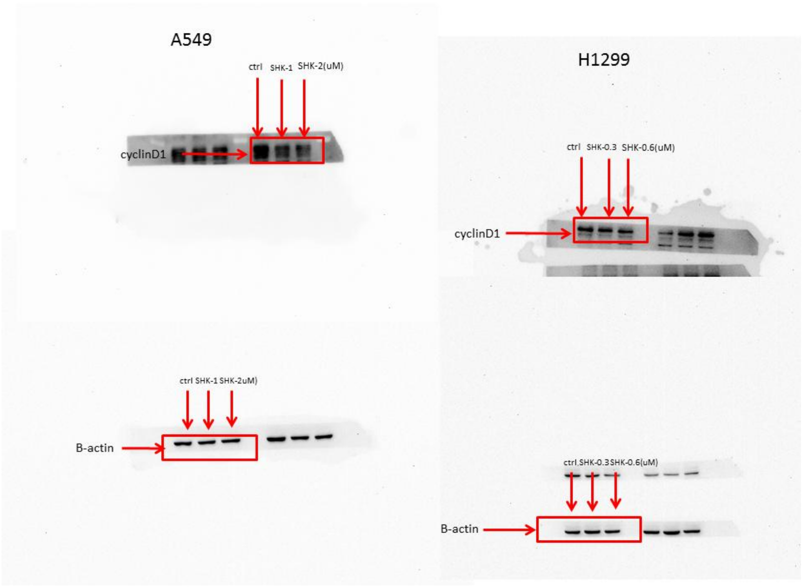

Original images in Figure1E

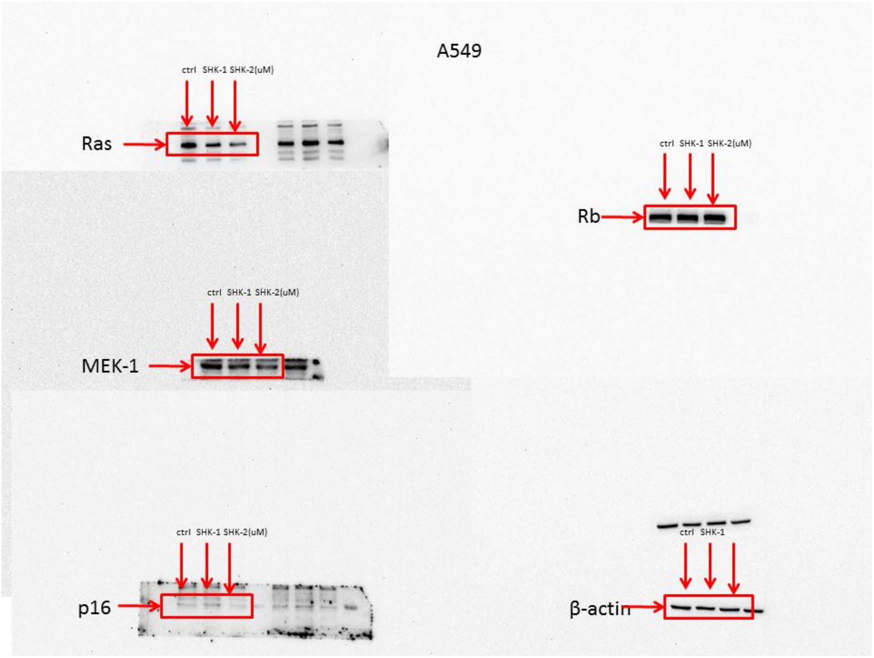

Original images in Figure3A

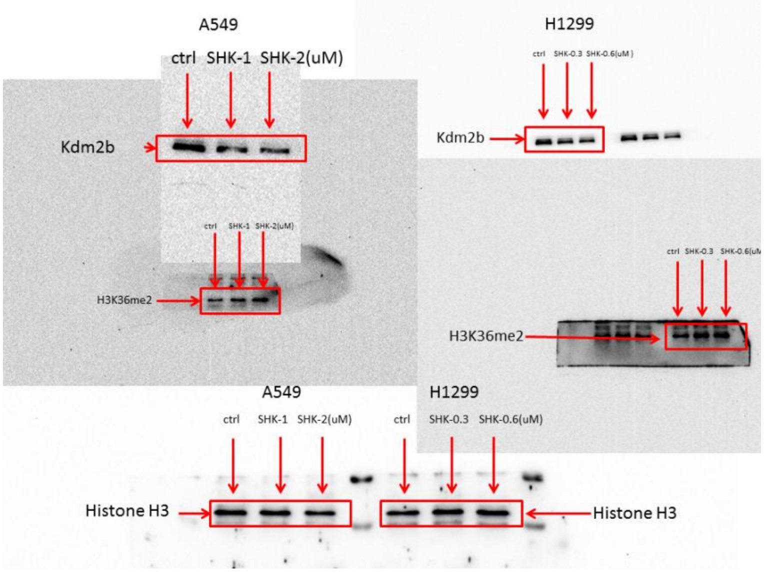

Original images in Figure3C

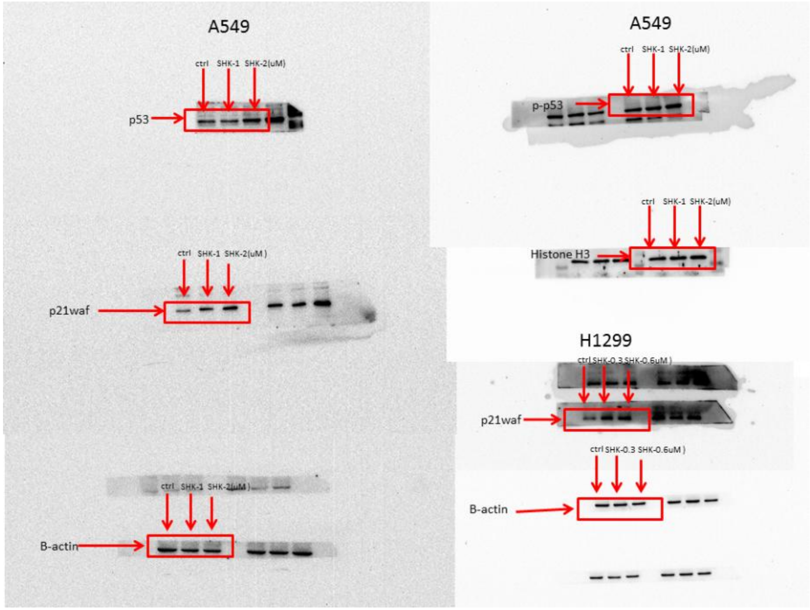

Original images in Figure3D and E

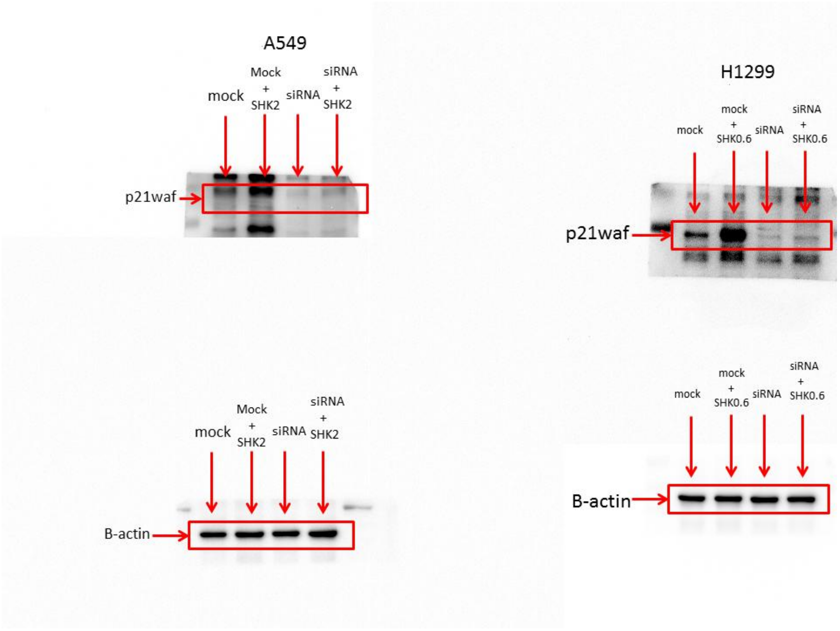

Original images in Figure3F

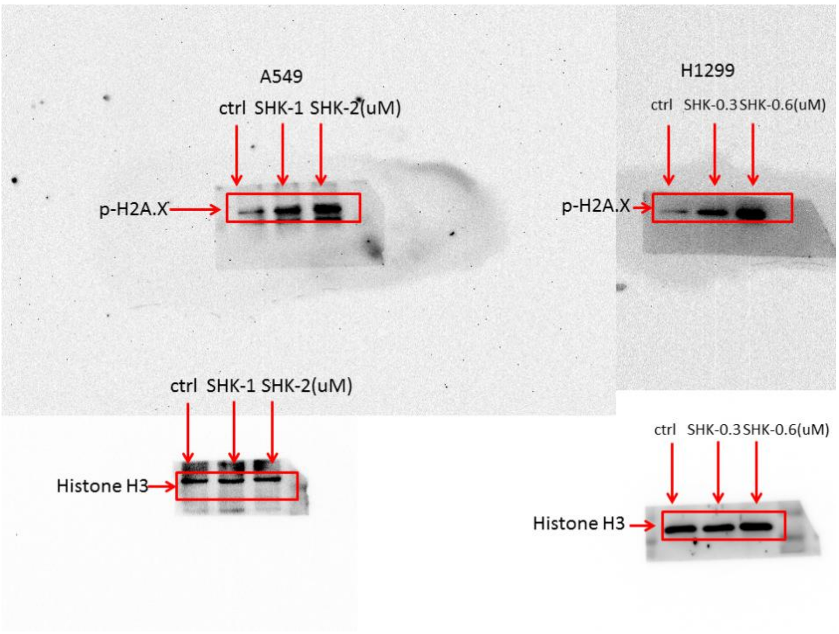

Original images in Figure3H

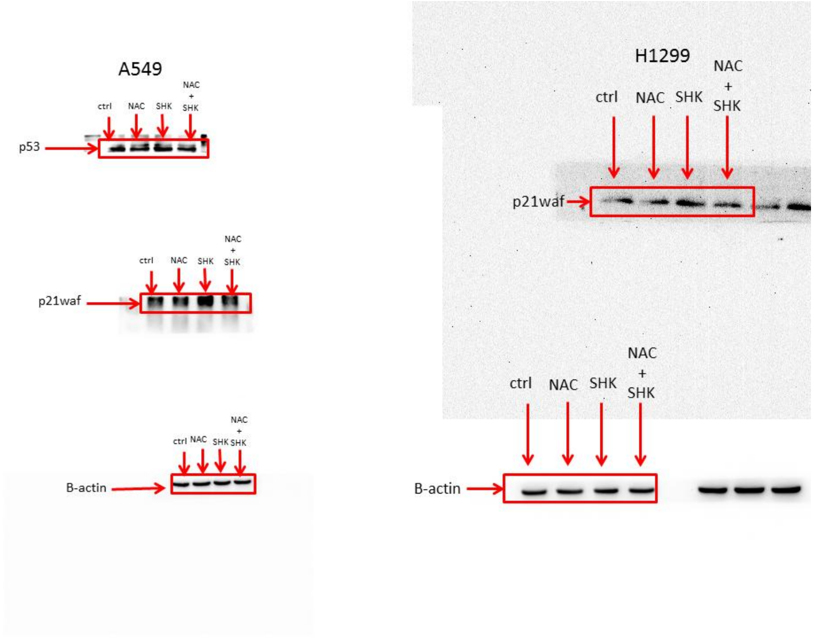

Original images in Figure4D

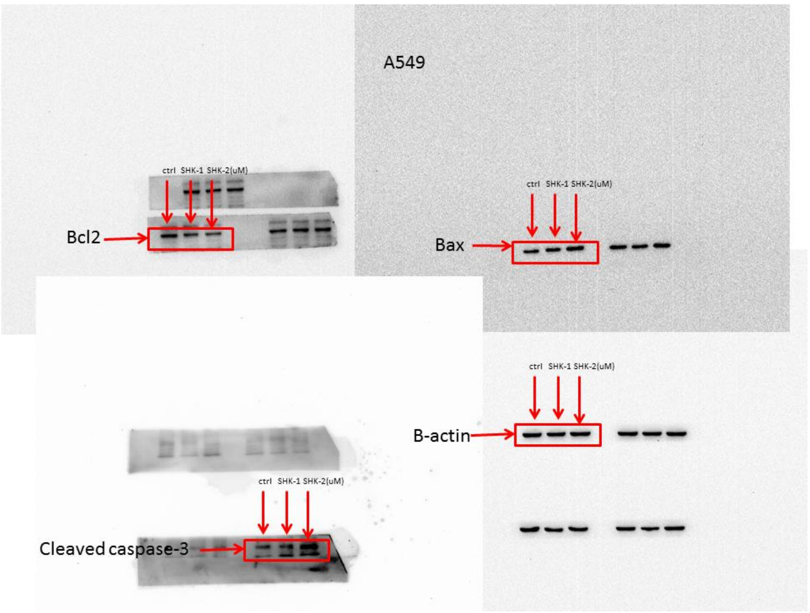

Original images in Figure5B

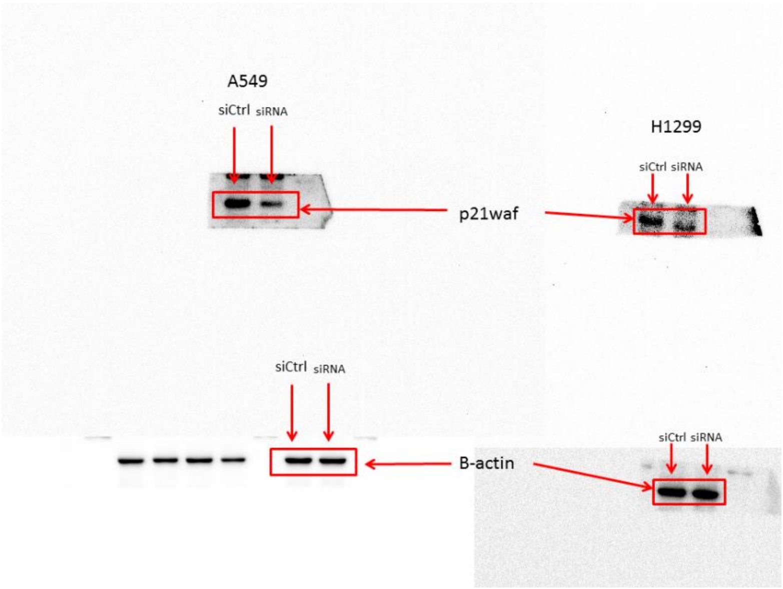

Original images in supplement Figure1

Review Certificate

To whom it may concern:

This menu certifies that one of our clients has contracted our academic editing service for the following file.

Order Number:
P-201803152079hmz
Word Count:
5427 words
Date of the review:
03/15/18 (MM/DD/YY)

The English review was conducted using a two-stage process, in which a junior editor first reviewed the file, and then a senior editor conducted a final and more thorough review. All of our editors are native English-speaking professionals.

Documents receiving this certification should be English-ready for publication; however, the author has the ability to accept or reject our suggestions and changes.

We would like to emphasize that our service targets grammar and language edits. We do not rewrite the documents from scratch. If you are dissatisfied with specific revisions, please contact service@essaystar.com.

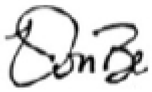

Essaystar Group

+1-208-975-4235
EssayStar, 93 S Jackson St, Seattle, WA 98104
